# Parallel auxin transport via PINs and plasmodesmata during the Arabidopsis leaf hyponasty response

**DOI:** 10.1007/s00299-023-03119-1

**Published:** 2023-12-20

**Authors:** Jiazhou Li, Jintao Yang, Yibo Gao, Ziyu Zhang, Chen Gao, Shaolin Chen, Johannes Liesche

**Affiliations:** 1 College of Life Sciences, Northwest A & F University, Yangling, 712100 China; 2 Biomass Energy Center for Arid and Semiarid Lands, Northwest A & F University, Yangling, 712100 China; 3 State Key Laboratory of Stress Biology for Arid Areas, Northwest A & F University, Yangling, 712100 China; 4 Institute for Molecular Physiology, University of Düsseldorf, 40225 Düsseldorf, Germany; 5 Institute of Biology, University of Graz, Schubertstraße 51, 8010 Graz, Austria

**Keywords:** Shade avoidance response, Neighbor detection, Auxin transporter, Diffusion, Auxin channeling, Leaf angle

## Abstract

**Key message:**

The leaf hyponasty response depends on tip-to-petiole auxin transport. This transport can happen through two parallel pathways: active trans-membrane transport mediated by PIN proteins and passive diffusion through plasmodesmata.

**Abstract:**

A plant’s ability to counteract potential shading by neighboring plants depends on transport of the hormone auxin. Neighbor sensing at the leaf tip triggers auxin production. Once this auxin reaches the abaxial petiole epidermis, it causes cell elongation, which leads to leaf hyponasty. Two pathways are known to contribute to this intercellular tip-to-petiole auxin movement: (i) transport facilitated by plasma membrane-localized PIN auxin transporters and (ii) diffusion enabled by plasmodesmata. We tested if these two modes of transport are arranged sequentially or in parallel. Moreover, we investigated if they are functionally linked. Mutants in which one of the two pathways is disrupted indicated that both pathways are necessary for a full hyponasty response. Visualization of PIN3-GFP and PIN7-GFP localization indicated PIN-mediated transport in parallel to plasmodesmata-mediated transport along abaxial midrib epidermis cells. We found plasmodesmata-mediated cell coupling in the *pin3pin4pin7* mutant to match wild-type levels, indicating no redundancy between pathways. Similarly, *PIN3*, *PIN4* and *PIN7* mRNA levels were unaffected in a mutant with disrupted plasmodesmata pathway. Our results provide mechanistic insight on leaf hyponasty, which might facilitate the manipulation of the shade avoidance response in crops.

**Supplementary Information:**

The online version contains supplementary material available at 10.1007/s00299-023-03119-1.

## Introduction

Access to light is one of the essential preconditions for plant growth and a key determinant of their productivity. Accordingly, plants have evolved various mechanisms to prevent shading by neighboring plants. One such mechanism is leaf hyponasty, i.e., the upward bending of the leaf (Franklin 2008). The primary signal for eliciting leaf hyponasty is a reduction in the red (R) to far-red (FR) light ratio resulting from selective R-light absorbance of the leaves of neighboring plants (Casal 2013). The low R:FR light condition is sensed by phytochromes in the cells of the leaf tip and triggers auxin synthesis (Küpers et al. 2020). Auxin moves from the leaf tip towards the petiole, where it triggers the expansion of cells on the basipetal side, thereby bending the leaf upwards (Ma and Li 2019).

Intercellular auxin transport can be facilitated by plasma membrane-localized transport proteins (Adamowski and Friml 2015). Auxin transporters of the *PIN-FORMED* family (*PINs*) have been implicated in the tip-to-petiole transport that triggers leaf hyponasty. There are several examples where polarized distribution of PIN proteins induces directional auxin transport in plant tissues (Ding et al. 2011; Zhang et al. 2013, 2017). In *Arabidopsis thaliana*, *PIN3* transcript abundance was induced by the low R:FR light condition (Pantazopoulou et al. 2017). Moreover, Arabidopsis mutant plants lacking PIN3, PIN4 and PIN7 showed a strongly reduced leaf hyponasty response to low R:FR conditions (Michaud et al. 2017; Pantazopoulou et al. 2017).

In addition to facilitation by membrane transporters, auxin can move intercellularly via plasmodesmata. These cell wall channels connect the cytosols of neighboring cells via a so-called cytoplasmic sleeve. With a width of about 3 nm, it enables concentration potential-driven diffusion of small molecules such as auxin (Rutschow et al. 2011; Liesche and Schulz 2013; Paterlini 2020; Band 2021). A high capacity for intercellular diffusion was found between endodermis cells, vascular parenchyma and especially the elongated cells of the leaf abaxial epidermis that run parallel to the mid-vein (Gao et al. 2020). Indeed, changes in the diffusion capacity of this pathway, as found in the Arabidopsis *gsl8* mutant, which is deficient in GLUCAN SYNTHASE LIKE 8 (GSL8), reduced tip-to-petiole auxin transport and leaf hyponasty (Gao et al. 2020). GSL8 activity leads to higher callose levels in the cell wall around plasmodesmata, which decreases the diameter of the cytosolic sleeve and, thereby, permeability. Accordingly, the *gsl8* mutant displays lower callose levels and higher plasmodesmata permeabilities. Despite this, the mutant’s tip-to-petiole auxin transport is reduced, because the high lateral diffusion capacity impairs the channeling effect that facilitates auxin diffusion along midrib epidermis cells in wild-type plants (Gao et al. 2020).

As described above, current data indicates that both, PIN- and plasmodesmata-mediated auxin transport, are required for a full shading-response, but how the two transport pathways are related is not known. Gao et al. calculated the auxin diffusion potential along epidermis cells above the midrib to be higher than what would be expected from PIN-mediated transport along epidermis or endodermis cells (Gao et al. 2020). At the same time, a major role for the PINs is suggested by the very strong reduction of hyponasty in the *pin3pin4pin7* mutant (Michaud et al. 2017; Pantazopoulou et al. 2017). One explanation could be that PINs are only involved in the transport towards the elongated cells, from where auxin moves through plasmodesmata. However, at least PIN3 has been located in the endodermis and the elongated cells of the adaxial epidermis (Park et al. 2019). Alternatively, PINs could be responsible for the lateral import of auxin into the elongated cells instead of facilitating basipetal transport. Previously, PIN3 in endodermal cells has been observed to relocate from the apical/basal plasma membrane to the lateral plasma membrane in Arabidopsis hypocotyls upon shading or auxin application (Keuskamp et al. 2010; Ding et al. 2011; Zhang et al. 2017).

Besides the question if PIN- and plasmodesmata-pathways are arranged sequentially or in parallel, another question is the interaction between the two pathways. Auxin has been shown to influence plasmodesmata permeability in Arabidopsis hypocotyls (Han et al. 2014). Moreover, PIN1 and PIN7 were part of an Arabidopsis plasmodesmata proteome (Fernandez-Calvino et al. 2011), indicating a potential link between PIN-mediated auxin transport and plasmodesmata function.

As described in the following, we investigated the relationship of PIN- and plasmodesmata-mediated auxin transport of the leaf hyponasty response, focusing on the abaxial epidermis cells that feature the higher cell coupling and better accessibility compared to endodermis cells. Clarifying the mechanism of neighbor-induced leaf hyponasty might be relevant for efforts to increase crop yield, as the response occurs in most crop species (Pantazopoulou et al. 2021). Indeed, the targeted suppression of shade avoidance responses has been identified as a promising strategy to increase planting densities without vegetative growth stimulation and, thereby, the improvement of yield per area (Carriedo et al. 2016).

## Results

### Reduced hyponasty responses in plasmodesmata- and *PIN*-mutants

**Fig. 1:**
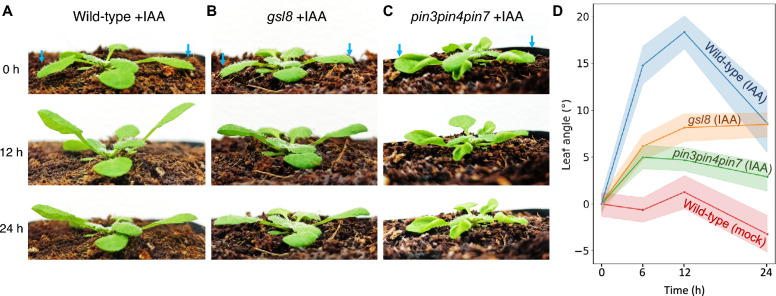
Hyponasty response in wild-type, *gsl8* and *pin3pin4pin7* plants. The auxin indole acetic acid (IAA) was applied to the tips of two leaves (arrows) of 21-day-old plants. **A**–**C** Images showing plants at the start of the experiment (0 h) and after 12 and 24 h. **D** Leaf angle change relative to the angle at 0 h. Shaded areas indicate 95% confidence intervals. Data for mock-treated wild-type plants were included as negative control. *N* = 7 (Wild-type mock), 36 (all other)

We triggered leaf hyponasty by application of the auxin indole acetic acid (IAA) to the leaf tip, a simple system that has been used in previous studies of the relevant transport and signaling pathways (Michaud et al. 2017; Pantazopoulou et al. 2017; Küpers et al. 2020). In addition, we conducted experiments in which the leaf tip is exposed to low R:FR light conditions to validate the results (Fig. S1). We measured leaf angle changes over time to compare the effects of pathway disruptions in the *gsl8* and *pin3pin4pin7* mutants. The effect was stronger in the *pin3pin4pin7* mutant, which displayed a maximum leaf angle change of about 25% of the wild-type level (Fig. 1A–D). The *gsl8* mutant leaves reached about 42% of the wild-type leaf angle (Fig. 1D). The results were not IAA-specific. Application of the auxin 1-naphthaleneacetic acid (NAA) instead of IAA caused similar reductions in leaf hyponasty in the *gsl8* and *pin3pin4pin7* mutants (Fig. S2). In control experiments, the application of water to wild-type plants (mock-treatment) indicated the diurnal and developmental effects on leaf angle to be in a limited range of below 5° within 24 h (Fig. 1D).

**Fig. 2:**
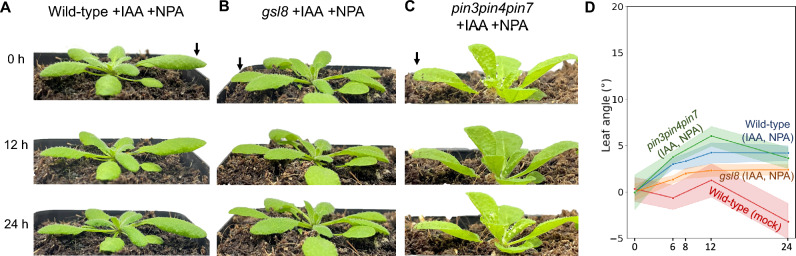
Effect of naphthylphthalamic acid (NPA) on the hyponasty response in wild-type, *gsl8* and *pin3pin4pin7* plants. NPA inhibits PIN auxin transporters. The auxin indole acetic acid (IAA) was applied to leaf tips of 21-day-old plants (arrows) after NPA was injected into the abaxial side of the leaf. **A**—**C** Images showing plants at the start of the experiment (0 h) and after 12 and 24 h. **D** Leaf angle change relative to the angle at 0 h. Data for mock-treated wild-type plants was included as negative control. Shaded areas indicate 95% confidence intervals. *N* = 7 (Wild-type mock), 14 (all other)

We probed the influence of PIN activity further by application of naphthylphthalamic acid (NPA), a competitive inhibitor of PIN-mediated auxin transport (Ung et al. 2022). Wild-type plants treated with NPA showed reduced leaf angle responses (Fig. 2A), with some similarity to the *pin3pin4pin7* mutants (Fig. 1D). The auxin-induced leaf angle increase of *gsl8* mutant plants treated with NPA was significantly lower than for wild-type plants, showing a maximum increase of only about 3 degree (Fig. 2B,D). Leaf angles of both types of plants treated with IAA and NPA were higher than in plants treated with neither IAA nor NPA (“Wild-type (mock)” in Fig. 2D). Treating the *pin3pin4pin7* mutant with NPA had no additional effect on the leaf angle response (Fig. 2C, D).

The experiments show that the combined disruption of PIN-mediated auxin transport and plasmodesmata-mediated auxin transport, as present in NPA-treated *gsl8* plants, leads to even stronger reduction of the hyponasty response than in plants where only one of the pathways is disrupted.

### PIN3 and PIN7 relocate to longitudinal interfaces of midrib epidermis cells

The results above led us to hypothesize a parallel arrangement of PIN- and plasmodesmata-pathways. To identify the positions of PIN transporters along the epidermal auxin transport pathway, we used plant lines expressing PIN-GFP-fusion proteins under their native promoters. Moreover, this allowed us to monitor redistribution of PINs during the hyponasty response. The transformed plants showed leaf hyponasty responses like wild-type plants, indicating that the GFP fusion does not influence PIN function (Fig. S3). Only images of PIN3-GFP and PIN7-GFP are shown. In plants transformed with PIN4-GFP we detected no specific fluorescence on our microscope system, presumably due to the generally low expression level of *PIN4* as analyzed below.

**Fig. 3:**
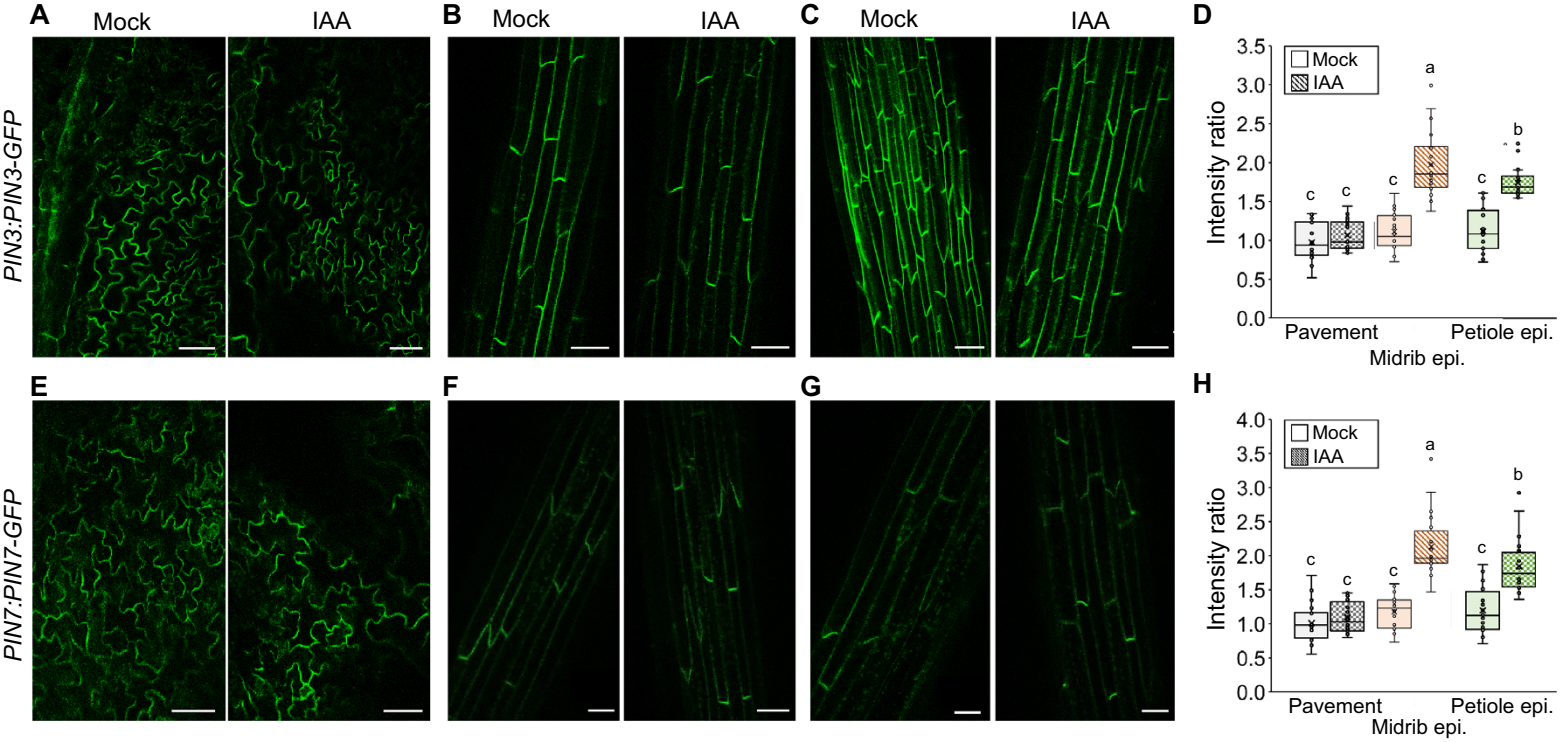
Localization of PIN-GFP fusion proteins two hours after application of water (mock) or IAA to the leaf tip. *PIN-GFP* constructs were expressed under native promoters. **A**–**C** Confocal microscopy images showing PIN3-GFP fluorescence in abaxial pavement epidermis cells (**A**), abaxial midrib epidermis cells (**B**) and abaxial petiole epidermis cells (**C**). **D** PIN3-GFP fluorescence intensity at the plasma membranes in line with the leaf axis divided by the intensity at the plasma membranes transverse to the leaf axis. **E**–**G** Confocal microscopy images showing PIN7-GFP fluorescence in abaxial pavement epidermis cells (**E**), abaxial midrib epidermis cells (**F**) and abaxial petiole epidermis cells (**G**). H PIN7-GFP fluorescence intensity at the plasma membranes in line with the leaf axis divided by the intensity at the plasma membranes transverse to the leaf axis. Scale bars: 50 µm. Similar letters on box plots indicate no significant difference (*P* > *0.05*) according to Student’s *t-test* (*N* = 20). Boxes represent quartiles, × represents mean, and bars indicate 95% confidence intervals. *N* = 20

PIN3-GFP and PIN7-GFP were both expressed in pavement and midrib cells of the leaf epidermis, as well as petiole epidermis cells (Fig. 3A-C, E–G). Auxin application influenced the intracellular distribution of PIN3-GFP and PIN7-GFP in the midrib and petiole epidermis cells (Fig. 3D, H). Two hours after auxin application to the leaf tip, the ratio of PIN3-GFP fluorescence at the longitudinal and transverse walls change from around 1 to around 2 (Fig. 3D), indicating that the concentration of PIN3-GFP at the longitudinal wall is twice as high as at the transverse wall. PIN3-GFP at the petiole epidermis showed a similar increase in intensity ratio from about 1 under control conditions to about 1.75 after auxin application (Fig. 3D). The results for PIN7-GFP resemble those of PIN3-GFP with strongly increased PIN7-GFP concentration at the longitudinal walls of midrib and petiole epidermis cells after auxin application (Fig. 3H). Similar relocation of PIN3-GFP and PIN7-GFP were observed after inducing the hyponasty response by lowering the R:FR light ratio at the leaf tip (Fig. S4).

**Fig. 4:**
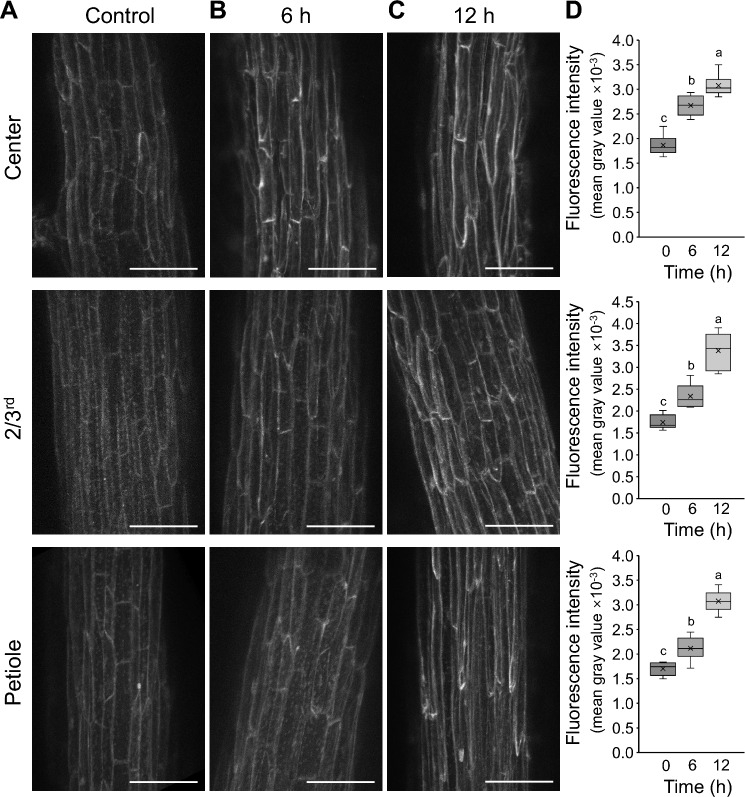
Auxin abundance in abaxial midrib epidermis cells visualized by the auxin-induced *DR5:GFP* reporter system. Leaf tips were exposed to low R:FR light conditions and GFP fluorescence detected at three positions: the center of the leaf (Center), about 2/3rd towards the leaf base (2/3rd) and petiole (Petiole). The mean fluorescence intensity of the epidermis cells in the field of view was measured. A-C Maximum projections of *DR5:GFP* fluorescence right before the low R:FR exposure (**A**), after 6 h (**B**) and after 12 h (**C**). Scale bars 50 µm. **D** Quantification of GFP fluorescence. Similar letters on box plots indicate no significant difference (*P* > *0.05*) according to Student’s *t-test* (*N* = 6). Boxes represent quartiles, × represents mean, and bars indicate 95% confidence intervals

Strong PIN3-GFP and PIN7-GFP expression in the leaf epidermis indicates the potential for auxin transport along this pathway. The presence of auxin in the midrib epidermis during the hyponasty response was investigated with the help of plants expressing the auxin-inducible promoter *DR5* coupled to a fluorescent reporter. Lowering the R:FR light ratio at the leaf tip led to increased *DR5*-driven reporter fluorescence in the midrib epidermis cells after 6 and 12 h (Fig. 4A–D). After 6 h, a strong increase in the leaf center was observed, while epidermis cells closer to the petiole and at the petiole showed minor increases (Fig. 4B,D). After 12 h, the epidermis at the lower leaf and at the petiole reached similarly high levels to the leaf center (Fig. 4C, D).

These results validate the hypothesis that PIN-mediated and plasmodesmata-mediated transport pathways are arranged in parallel along the midrib epidermis cells.

### Plasmodesmata structure is unchanged in *pin3pin4pin7* mutant

**Fig. 5:**
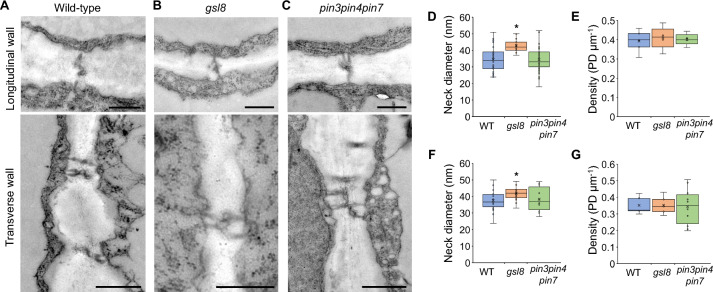
Plasmodesmata in adaxial midrib epidermis cells in wild-type plants and *gsl8* and *pin3pin4pin7* mutants. **A**–**C** Transmission electron micrographs showing the structure of plasmodesmata at the longitudinal and transverse walls relative to the leaf axis in wild-type (**A**), *gsl8* mutant (**B**) and *pin3pin4pin7* mutant (**C**). Scale bars 500 nm. **D**, **E** Neck diameter (**D**) and density (**E**) of plasmodesmata (PD) at the longitudinal walls. **F**, **G** Neck diameter (**F**) and density (**G**) at the transverse walls. *WT* wild-type. Asterisks indicate significant difference according to Student’s *t-test* (*P* < *0.05*). Boxes represent quartiles, × represents mean, and bars indicate 95% confidence intervals. Sample numbers for wild-type, *gsl8*, *pin3pin4pin7*: 22, 14, 10 (**D**), 5, 5, 5 (**E**), 35, 13, 46 (**F**), 5, 5, 10 (**G**)

The parallel arrangement of transport pathways could enable functional redundancy. To investigate if the lack of PIN proteins in the *pin3pin4pin7* mutant influenced the plasmodesmata-mediated transport pathway, we analyzed plasmodesmata structure and function. Transmission electron microscopy of plasmodesmata in the longitudinal and transverse walls of midrib epidermis cell revealed no major differences in plasmodesmata structure, for example with regard to branching, between wild-type, *gsl8* and *pin3pin4pin7* mutant plants (Fig. 5A–C). As expected, neck diameters of plasmodesmata in the longitudinal and transverse walls of *gsl8* mutants were increased (Fig. 5D, F). *pin3pin4pin7* mutants showed no significant changes in neck diameter or plasmodesmata density compared to wild-type plants at both sides of the cells (Fig. 5D–G). This result does not indicate pathway redundancy, since the compromised PIN-mediated auxin transport in the *pin3pin4pin7* mutant did not lead to plasmodesmata that would enable increased auxin diffusion capacity.

### Intercellular permeability in *pin3pin4pin7* resembles wild-type

**Fig. 6:**
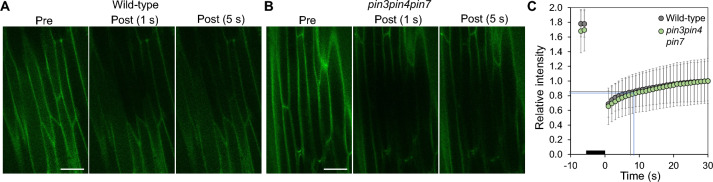
Cell coupling of abaxial midrib epidermis cells in wild-type and *pin3pin4pin7* mutant plants. Cell coupling was quantified using the fluorescence redistribution after photobleaching (FRAP) method. **A**, **B** Confocal images showing fluorescence of a small cytosolic tracer molecule before photobleaching (Pre) and after photobleaching of a central quadratic area (Post 1 s, Post 5 s) in wild-type (**A**) and *pin3pin4pin7* mutant (**B**). Scale bars: 50 µm. C Relative fluorescence intensity in the bleached area. Horizontal and vertical lines indicate the half-time of recovery. Error bars indicate standard deviation. *N* = 13 (wild-type), 12 (*pin3pin4pin7*)

In addition to the structural analysis, we tested the function of plasmodesmata by fluorescence redistribution after photobleaching (FRAP) experiments on intact leaves of wild-type and *pin3pin4pin7* plants. After bleaching a small fluorescent molecule in a few midrib-epidermis cells, we monitored recovery of fluorescence in these cells due to the redistribution from neighboring cells. A shorter half-life of this redistribution indicates faster intercellular movement and, thereby, a larger capacity for plasmodesmata-mediated transport. No significant differences between wild-type and *pin3pin4pin7* were observed (Fig. 6A–C). The half-times of fluorescence recovery were 7.5 s (standard deviation 2.3 s) for wild-type and 8.4 s (standard deviation 2.6 s) for *pin3pin4pin7*. Previously, we found no influence of auxin application on midrib epidermis cell coupling in wild-type leaves (see Fig. S2G in Gao et al. 2020). Similarly, FRAP experiments on *pin3pin4pin7* mutant leaves eight hours after auxin application did not differ significantly from control conditions. The half-times of fluorescence recovery on auxin-treated plants were 7.3 s (standard deviation 1.9 s) for wild-type and 7.8 s (standard deviation 2.2 s) for *pin3pin4pin7*. Like the TEM results above, results of the FRAP experiments indicate no influence of reduced PIN-mediated auxin transport on plasmodesmata-mediated auxin diffusion capacity.

### *PIN* mRNA levels are not increased in *gsl8* mutant

**Fig. 7:**
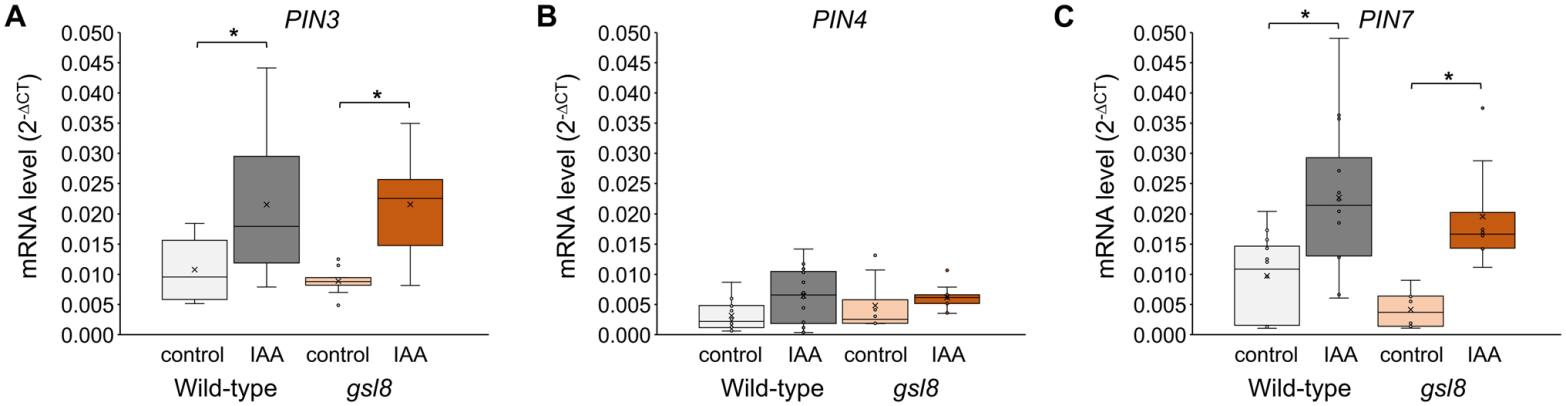
mRNA levels of *PIN* transporters in leaves of wild-type and *gsl8* mutant plants. mRNA levels were measured by qPCR in untreated plants (control) or plants on which the auxin IAA was applied to the leaf tip (IAA). **A** *PIN3* mRNA level. **B** *PIN4* mRNA level, **C** *PIN7* mRNA level. Boxes represent quartiles, × represents mean, and bars equal 95% confidence intervals. Asterisks indicate significant differences (*P* < *0.05*) as tested by *t-test* (*N* = 6)

We further tested if *PINs* are upregulated in *gsl8* mutant plants with their reduced tip-to-petiole auxin diffusion efficiency. Transcript abundance of *PIN3*, *PIN4* and *PIN7* was quantified in wild-type and *gsl8* plants. Two hours after application of auxin to the leaf tip, *PIN3* and *PIN7* mRNA levels strongly increased in leaves of wild-type plants (Fig. 7A, C), mirroring results from our earlier measurements (Fig. S2F in Gao et al. 2020). *PIN4* was expressed at a low level and showed no significant response to auxin application (Fig. 7B). *PIN3*, *PIN4* and *PIN7* mRNA levels in the *gsl8* mutant matched those in wild-type plants, including the auxin-dependent increase in *PIN3* and *PIN7* mRNA abundance (Fig. 7A–C). This result does not indicate a link between disruption of the auxin diffusion pathway and the transcriptional regulation of relevant *PINs*.

## Discussion

PIN proteins have long been seen as the main facilitators of tip-to-petiole auxin transport during the leaf hyponasty response (Michaud et al. 2017; Pantazopoulou et al. 2017). Several of our experiments confirm their central role. As seen before, leaf hyponasty was strongly reduced in the *pin3pin4pin7* triple mutant. Transcript levels of *PIN3* and *PIN7* strongly increased after triggering of the hyponasty response. Moreover, PIN3 and PIN7 were found to be present in midrib and petiole epidermis cells, in which their concentration at the longitudinal walls increased upon triggering of the hyponasty response. These results suggest that auxin transport is facilitated by PIN3 and PIN7 all the way from the leaf tip to the petiole. PIN-mediated and plasmodesmata-mediated transport pathways appear to be running in parallel in the midrib and petiole epidermis cells. A similar arrangement could be expected for endodermis cells, where the situation with high longitudinal cell wall permeability (Gao et al. 2020) and strong *PIN3* expression (Park et al. 2019), especially at longitudinal cell interfaces (Küpers et al. 2020), resembles that of the midrib epidermis.

Does a parallel arrangement of PIN- and plasmodesmata-mediated auxin pathways imply redundancy and interaction? Our data provides no evidence for this. Plasmodesmata function remained unchanged in the midrib and petiole epidermis cells of the *pin3pin4pin7* mutant. Similarly, *PIN* transcript levels were not changed by the disruption of the plasmodesmata pathway in the *gsl8* mutant. This lack of compensation suggests that there are mechanical issues that prevent the malfunction of one pathway to influence the capacity of the other pathway. For example, plasmodesmata structure and density might not be influenced because of their prescribed role during leaf development (Burch-Smith et al. 2011). Another possibility is a limitation of feedback signaling within the hyponasty response. Regulation of PINs by positive feedback loops has been described for the establishment of local auxin maxima in meristems (Wang and Jiao 2018). However, the long tip-to-petiole distance of at least several millimeters might hinder coordination (Renton et al. 2012). Moreover, the high overall plasmodesmata permeability of leaf cells in the *gsl8* mutant (Gao et al. 2020) means that auxin is likely to disperse from the tip. This could prevent a buildup of auxin at the tip, which would be the most likely cue for the upregulation of auxin transport (Renton et al. 2012). Measurements of local auxin concentrations could be used to test this hypothesis. Auxin concentration should also be measured in the petiole of *gsl8* mutant plants, as it cannot currently be excluded that it is affected by R:FR-independent processes. However, a major influence on the hyponasty response would be surprising as the timing of the *gsl8* mutant’s hyponasty response agreed with the results of tip-to-petiole auxin transport monitoring (Gao et al. 2020).

It should be noted that we did not test transport activity of PIN3 and PIN7 in the *gsl8* mutant. Reduced callose synthesis could have induced PIN activity through post-transcriptional modifications, such as phosphorylation. Indeed, links between callose synthesis and salicylic acid production (Zhong et al. 2021) and between salicylic acid and PIN activity (Tan et al. 2020) have been recently established, although not regarding leaf hyponasty. Auxin transport assays might be adapted to test the influence of callose synthase on PIN3 and PIN7 activity (Barbosa et al. 2018). Another aspect that should be explored is the effect of reduced plasmodesmata permeability on PIN-mediated auxin transport. Plasmodesmata are not blocked in the *gsl8* mutant, where tip-to-petiole auxin diffusion is compromised by the missing channeling effect of interface-specific plasmodesmata regulation (Gao et al. 2020). The effect of reduced plasmodesmatal diffusion capacity could be tested by expressing the *icals3m*-module in mesophyll epidermis and endodermis cells. This would enable the induction of callose synthesis, and thereby the reduction of plasmodesmata permeability, at the relevant interfaces (Liu et al. 2017; Li et al. 2022).

In conclusion, our work showed that active (PIN-mediated) and passive (plasmodesmata-mediated) auxin transport both contribute to the hyponasty response. Both transport systems work in parallel in channeling auxin along the midrib epidermis cells. Future measurements of transport rates for both pathways at high spatial and temporal resolution will further clarify the relative contributions of the two pathways.

## Methods

### Plant material and growth conditions

Arabidopsis plants were soil-grown in growth chambers under a 16-h light/8-h dark regime, with 90 μmol photon·m^−2^·s^−1^ light intensity and 22 °C. We used the soil Nature’s Gro (Miracle-Gro, OH, USA), which contained total nitrogen at 0.068%, available phosphate (P_2_O_5_) at 0.027% and soluble potash (K_2_O) at 0.036%. The measured pH ranged between 5.5 and 6.5. Seeds were imbibed for 3 d in dark at 4 °C, then sterilized in 75% alcohol for 2 min and 10% (v/v) sodium hypochlorite for 10 min and washed 5 times with sterilized water. All Arabidopsis plants used in this study belong to the accession Col-0. Seeds of the *gsl8* mutant were purchased from the Nottingham Arabidopsis Stock Center (stock number N665827). Seeds of *pin3pin4pin7* were obtained from the Friml lab (Blilou et al. 2005). Primers used for genotyping are listed in Table S1.

### Evaluation of hyponasty response

The standard procedure for measuring the leaf angle change was to apply 1 µL of 100 µM IAA to the tip of the largest one or two leaves, similar to what has been described by Gao et al. (2020). Mock treatment was conducted with 1 µL of distilled water. An IAA stock solution was prepared by dissolving 35.04 mg of IAA powder in 2 mL ethanol. NAA was applied, like IAA, as a drop of 1 µL of 100 µM NAA. NAA stock solution was prepared by dissolving 37.24 mg of NAA powder in 2 mL ethanol. NPA was injected into the abaxial leaf epidermis using a 1 mL syringe without needle at a concentration of 50 µM. An NPA stock solution was produced by dissolving 5 mg NPA powder in 3.43 mL ethanol. Stock solutions were stored at  – 20 °C. Working solutions were freshly prepared for each set of experiments. The plants were at the rosette stage, 28 days old. Treatments were always performed at 11 AM and photographs taken after 6, 11, 12 and 24 h in a fixed setup. Leaf angles were calculated from photographs. The alternative procedure for eliciting hyponasty was to lower the R:FR light ratio at the leaf tip similar to the procedure described by Michaud et al. (2017). A LED 730 nm light source was adjusted with the help of aluminum foil to illuminate the leaf tip. Photographs were taken at the beginning of the experiment and after 24 h.

### PIN-GFP localization

Plants stably expressing *PIN3::PIN3-GFP* (Zádníková et al. 2010), *PIN4::PIN4-GFP* (Vieten et al. 2005) and *PIN7::PIN7-GFP* (Blilou et al. 2005) constructs were grown for 3 weeks. GFP fluorescence was recorded using a confocal laser-scanning module (Thorlabs, Newton, NJ, USA) mounted on a Leica DMi8 microscope frame (Leica Microsystems, Wetzlar, Germany) with excitation at 488 nm and emission detection with a 494–524 nm band-pass filter. Leaves were cut off at the petiole with a razorblade two hours after auxin application to the leaf tip or exposure to low R:FR light conditions. Leaves were gently placed on a slide and analyzed immediately using a 20 × water-immersion objective. Z-scans were performed to capture many in-focus cells in these thick uneven samples. ImageJ (Schindelin et al. 2012) was used to measure the mean fluorescence intensity value of single cells or single plasma membrane interfaces within cells. For that purpose, regions of interest were drawn around cells or around the specific interface. In case of plasma membrane interfaces the region of interest had a fixed width of 5 µm.

### Visualization of *DR5* activity

Plants stably expressing *DR5:GFP* (Ottenschläger et al. 2003) were grown for 3 weeks. GFP fluorescence was detected by confocal microscopy as described above. Leaves were cut off at the petiole with a razorblade either right before the light was modulated (control) or six or twelve hours after the start of low R:FR exposure. Leaves were gently placed on a slide and analyzed immediately using a 20 × water-immersion objective. Z-scans were performed to capture many in-focus cells at the abaxial midrib epidermis at three positions: in the center of the leaf, about 2/3rd from the leaf tip and at the petiole. The mean fluorescence intensity value of a 180 × 70 µm area, containing about ten cells, was determined and served as indicator of *DR5*-activity. Intracellular background fluorescence was visible in some images. However, it did not affect measurements as it occurred to a similar degree across the images of each sample type.

### Analysis of plasmodesmata structure and density

Plasmodesmata were analyzed on 3-week-old wild-type, *gsl8* and *pin3pin4pin7* plants. Leaves were cut into strips about 4–5 mm long and 2 mm wide, and an incision was made on one corner of the abaxial side to be able to distinguish adaxial and abaxial surfaces. The samples were immediately placed in a 4% glutaraldehyde solution under dark conditions for fixation. Then they were rinsed three times with phosphate-buffered saline (PBS, pH 7.2) for 15 min, fixed with osmic acid for 2–4 h, and rinsed three more times with PBS. Gradient dehydration was performed successively in different concentrations of ethanol (30%, 50%, 70%, 90%, 100%) twice for 10 min. Gradient osmosis was performed in alcohol: LR-White mixtures: 3:1 for 2 h, 1:1 for 8 h, 1:3 for 12 h, followed by incubation in LR-White twice for 24 h. After embedding in rubber molds, the polymerization was carried out at 55 °C for 48 h. The excess polymerized LR-White above the sample was trimmed off. Semi-thin Sects. (0.5 to 1 µm thickness) were cut using an ultramicrotome (UC7, Leica Microsystems) until the target position (abaxial epidermis) was reached. Here, samples were ultrathin sectioned (70 nm thickness) and sections transferred to copper meshes. Sections on the copper meshes were stained with 2% uranyl acetate for 20 min, and the surface dye residue was thoroughly rinsed with pure water. Then, sections were stained with lead citrate for 15 min, and the dye residue was rinsed. The stained samples were observed under a transmission electron microscope (TECNAI G2 SPIRIT BIO, FEI Hillsboro, OR, USA). Images were taken at a smaller magnification to facilitate the calculation of the plasmodesmata density, and at larger magnification to investigate plasmodesmata structure. ImageJ was used to measure plasmodesmata neck diameter and density following Liesche et al. (2019).

### Analysis of cell coupling

Leaves of three-week-old wild-type and *pin3pin4pin7* mutant plants were used for FRAP experiments. The cytosolic tracer carboxyfluorescein diacetate (cFDA, ThermoFisher, Waltham MA, USA) was used at a concentration of 50 µM (diluted in PBS buffer, pH 7.2). Six hours after the application of auxin to the leaf tip, leaves were gently cut at the petiole and soaked in cFDA solution for 20 min. After washing with PBS, leaves were mounted on microscope slides and analyzed by confocal microscopy employing a 20 × water-immersion objective. cFDA fluorescence was excited at 488 nm and emission detected with a 494 to 524 nm band-pass filter. A location at the center of the abaxial midrib epidermis covering about 25% of image area was selected for bleaching. Fluorescence in this area was bleached by high laser light intensity and zoom-in for 10–20 s. Fluorescence recovery was monitored for about 1 min. ImageJ was used to quantify the fluorescence intensity in the target region before and after bleaching. Nonlinear regression curve fitting on the post-bleaching data and determination of half-times was performed with GraphPad Prism 8 (GraphPad Software, San Diego, CA, USA).

### Quantification of *PIN* mRNA levels

*PIN* mRNA levels were measured on leaves of 3-week-old wild-type and *gsl8* mutant plants. Leaf samples were taken 2 h after application of IAA to the leaf tip as described above. Total RNA from the leaf samples was extracted using Trizol (Invitrogen, Carlsbad, CA, USA) according to the manufacturer’s instructions. The quantity and quality of RNA were assessed spectroscopically (Nanodrop ND-2000, Thermo Scientific, MA, USA). The first-strand cDNA was synthesized with the EasyScript One-Step gDNA Removal and cDNA Synthesis SuperMix (Transgen, Beijing, China). mRNA levels of *PIN3* (AT1G70940), *PIN4* (AT2G01420) and *PIN7* (AT1G23080) were quantified by qPCR using the CFX Connect Real Time System (Bio-Rad, Feldkirchen, Germany) with TransStart Tip Green qPCR SuperMix (Transgen) according to the manufacturer’s instructions. mRNA levels were normalized to reference gene *UBQ10* (AT4G05320). mRNA levels were calculated using the 2^−ΔCt^ method. Primer sequences are listed in Table S2.

### Statistical methods

To evaluate the significance of pairwise differences between samples, P values were calculated using Student’s *t-test* with significance indicated by *P* < *0.05*. Sample numbers are provided in the figure legends.

### Supplementary Information

Below is the link to the electronic supplementary material.

Supplementary file1 (DOCX 1269 KB)

## Data Availability

The published article includes all datasets generated or analyzed during this study. The authors will be happy to share raw data on request.

## References

[CR1] Adamowski M, Friml J (2015). PIN-dependent auxin transport: action, regulation, and evolution. Plant Cell.

[CR2] Band LR (2021). Auxin fluxes through plasmodesmata. New Phytol.

[CR3] Barbosa I, Hammes UZ, Schwechheimer C (2018). Activation and polarity control of PIN-FORMED auxin transporters by phosphorylation. Trends Plant Sci.

[CR4] Blilou I, Xu J, Wildwater M, Willemsen V, Paponov I, Friml J, Heidstra R, Aida M, Palme K, Scheres B (2005). The PIN auxin efflux facilitator network controls growth and patterning in Arabidopsis roots. Nature.

[CR5] Burch-Smith TM, Stonebloom S, Xu M, Zambryski PC (2011). Plasmodesmata during development: re-examination of the importance of primary, secondary, and branched plasmodesmata structure versus function. Protoplasma.

[CR6] Carriedo LG, Maloof JN, Brady SM (2016). Molecular control of crop shade avoidance. Curr Opin Plant Biol.

[CR7] Casal JJ (2013). Photoreceptor signaling networks in plant responses to shade. Annu Rev Plant Biol.

[CR8] Ding Z, Galvan-Ampudia CS, Demarsy E, Langowski L, Kleine-Vehn J, Fan Y, Morita MT, Tasaka M, Fankhauser C, Offringa R, Friml J (2011). Light-mediated polarization of the pin3 auxin transporter for the phototropic response in Arabidopsis. Nat Cell Biol.

[CR9] Fernandez-Calvino L, Faulkner C, Walshaw J, Saalbach G, Bayer E, Benitez-Alfonso Y, Maule A (2011). Arabidopsis Plasmodesmal Proteome. PLoS ONE.

[CR10] Franklin KA (2008). Shade avoidance. New Phytol.

[CR11] Gao C, Liu X, De Storme N, Jensen KH, Xu Q, Yang J, Liu X, Chen S, Martens HJ, Schulz A, Liesche J (2020). Directionality of plasmodesmata-mediated transport in Arabidopsis leaves supports auxin channeling. Curr Biol.

[CR12] Han X, Hyun TK, Zhang M, Kumar R, Koh EJ, Kang BH, Lucas WJ, Kim JY (2014). Auxin-callose-mediated plasmodesmal gating is essential for tropic auxin gradient formation and signaling. Dev Cell.

[CR13] Keuskamp DH, Pollmann S, Voesenek LA, Peeters AJ, Pierik R (2010). Auxin transport through PIN-FORMED3 (PIN3) controls shade avoidance and fitness during competition. Proc Natl Acad Sci USA.

[CR14] Küpers JJ, Oskam L, Pierik R (2020). Photoreceptors regulate plant developmental plasticity through auxin. Plants-Basel.

[CR15] Li M, Wang M, Lin Q, Wang M, Niu X, Cheng J, Xu M, Qin Y, Liao X, Xu J, Wu S (2022). Symplastic communication in the root cap directs auxin distribution to modulate root development. J Integr Plant Biol.

[CR16] Liesche J, Schulz A (2013). Modeling the parameters for plasmodesmal sugar filtering in active symplasmic phloem loaders. Front Plant Sci.

[CR17] Liesche J, Gao C, Binczycki P, Andersen SR, Rademaker H, Schulz A, Martens HJ (2019). Direct comparison of leaf plasmodesma structure and function in relation to phloem-loading type. Plant Physiol.

[CR18] Liu Y, Xu M, Liang N, Zheng Y, Yu Q, Wu S (2017). Symplastic communication spatially directs local auxin biosynthesis to maintain root stem cell niche in Arabidopsis. Proc Natl Acad Sci USA.

[CR19] Ma L, Li G (2019). Auxin-dependent cell elongation during the shade avoidance response. Front Plant Sci.

[CR20] Michaud O, Fiorucci AS, Xenarios I, Fankhauser C (2017). Local auxin production underlies a spatially restricted neighbor-detection response in Arabidopsis. Proc Natl Acad Sci USA.

[CR21] Ottenschlager I, Wolff P, Wolverton C, Bhalerao RP, Sandberg G, Ishikawa H, Evans M, Palme K (2003). Gravity-regulated differential auxin transport from columella to lateral root cap cells. Proc Natl Acad Sci USA.

[CR22] Pantazopoulou CK, Bongers FJ, Kupers JJ, Reinen E, Das D, Evers JB, Anten N, Pierik R (2017). Neighbor detection at the leaf tip adaptively regulates upward leaf movement through spatial auxin dynamics. Proc Natl Acad Sci USA.

[CR23] Pantazopoulou CK, Bongers FJ, Pierik R (2021). Reducing shade avoidance can improve Arabidopsis canopy performance against competitors. Plant Cell Environ.

[CR24] Park YJ, Lee HJ, Gil KE, Kim JY, Lee JH, Lee H, Cho HT, Vu LD, De Smet I, Park CM (2019). Developmental programming of thermonastic leaf movement. Plant Physiol.

[CR25] Paterlini A (2020). Uncharted routes: exploring the relevance of auxin movement via plasmodesmata. Biol Open.

[CR26] Renton M, Hanan J, Ferguson BJ, Beveridge CA (2012). Models of long-distance transport: how is carrier-dependent auxin transport regulated in the stem?. New Phytol.

[CR27] Rutschow HL, Baskin TI, Kramer EM (2011). Regulation of solute flux through plasmodesmata in the root meristem. Plant Physiol.

[CR28] Schindelin J, Arganda-Carreras I, Frise E, Kaynig V, Longair M, Pietzsch T, Preibisch S, Rueden C, Saalfeld S, Schmid B, Tinevez JY, White DJ, Hartenstein V, Eliceiri K, Tomancak P, Cardona A (2012). Fiji: an open-source platform for biological-image analysis. Nat Methods.

[CR29] Tan S, Abas M, Verstraeten I, Glanc M, Molnar G, Hajny J, Lasak P, Petrik I, Russinova E, Petrasek J, Novak O, Pospisil J, Friml J (2020). Salicylic acid targets protein phosphatase 2a to attenuate growth in plants. Curr Biol.

[CR30] Ung KL, Winkler M, Schulz L, Kolb M, Janacek DP, Dedic E, Stokes DL, Hammes UZ, Pedersen BP (2022). Structures and mechanism of the plant PIN-formed auxin transporter. Nature.

[CR31] Vieten A, Vanneste S, Wisniewska J, Benkova E, Benjamins R, Beeckman T, Luschnig C, Friml J (2005). Functional redundancy of PIN proteins is accompanied by auxin-dependent cross-regulation of pin expression. Development.

[CR32] Wang Y, Jiao Y (2018). Auxin and above-ground meristems. J Exp Bot.

[CR33] Zadnikova P, Petrasek J, Marhavy P, Raz V, Vandenbussche F, Ding Z, Schwarzerova K, Morita MT, Tasaka M, Hejatko J, Van Der Straeten D, Friml J, Benkova E (2010). Role of PIN-mediated auxin efflux in apical hook development of Arabidopsis thaliana. Development.

[CR34] Zhang KX, Xu HH, Yuan TT, Zhang L, Lu YT (2013). Blue-light-induced PIN3 polarization for root negative phototropic response in Arabidopsis. Plant J.

[CR35] Zhang Y, Yu Q, Jiang N, Yan X, Wang C, Wang Q, Liu J, Zhu M, Bednarek SY, Xu J, Pan J (2017). Clathrin regulates blue light-triggered lateral auxin distribution and hypocotyl phototropism in Arabidopsis. Plant Cell Environ.

[CR36] Zhong Q, Hu H, Fan B, Zhu C, Chen Z (2021). Biosynthesis and roles of salicylic acid in balancing stress response and growth in plants. Int J Mol Sci.

