# Supplementary material for: Parallel auxin transport via PINs and plasmodesmata during the Arabidopsis leaf hyponasty response
Source: Plant Cell Rep. 2023 Dec 20;43(1):4. doi: 10.1007/s00299-023-03119-1 (PMC10733227; doi:10.1007/s00299-023-03119-1)
Supplement: Supplementary file 1 — Supplementary file1 (DOCX 1269 KB) [file 299_2023_3119_MOESM1_ESM.docx]

**Supplemental Material**

for

**Parallel auxin transport via PINs and plasmodesmata during the Arabidopsis leaf hyponasty response**

Jiazhou Li, Jintao Yang, Yibo Gao, Ziyu Zhang, Chen Gao, Shaolin Chen, Johannes Liesche

This PDF file includes:

Table S1
Table S2
Figures S1 to S4

**Supplemental tables**

Table S1. Primers used for genotyping of mutant lines.

| Primer | Sequence |
| --- | --- |
| MG_pin3-5_F | CCCATCCCCAAAAGTAGAGTG |
| MG_pin3-5_F | ATGATACACTGGAGGACGACG |
| pin4-3_Fw | AACCGGTACGGGTGTTTCAACTA |
| pin4-3_Rev | GCCATTCCAAGACCAGCATCT |
| MG_pin7-1_F | AAATCCGATCAAGGCGGTG |
| MG_pin7-1_R | CGTCGAATTTCCGCAAGC |
| GSL8_F | ATATGCTGCGATGTTTTCACC |
| GSL8_R | AACGAAAAGGGAATTCAAAGC |

Table S2. Primers used for the quantification of *PIN* mRNA levels.

| Primer | Sequence |
| --- | --- |
| PIN3 forward | ACCAACAATCCTTACGCAAT |
| PIN3 reverse | AGGAATCCCCATAACAAGAGT |
| PIN4 forward | CTACGCAGGTTCTCTAATGGTC |
| PIN4 reverse | TCGGATTCAACTTTAAACGAA |
| PIN7 forward | GTACCCAGCTCCTAATCCG |
| PIN7 reverse | TTCCGACTTGTTCATTGGCTC |
| UBQ10 forward | GGCCTTGTATAATCCCTGATGAATAAG |
| UBQ10 reverse | AAAGAGATAACAGGAACGGAAACA |

**Supplemental figures**


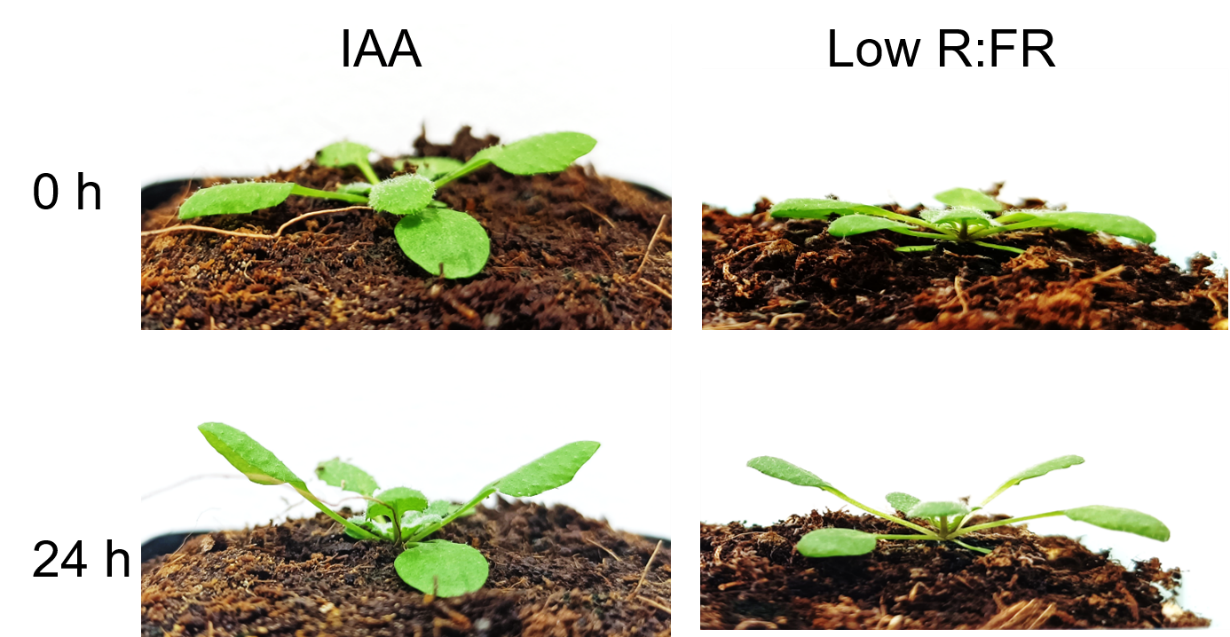


**Fig. S1: Hyponasty of wild-type plants after application of auxin (IAA) to the leaf tip or exposure to lower R:FR light ratio.** Photographs indicating similar leaf angle changes resulting from the two procedures.

**
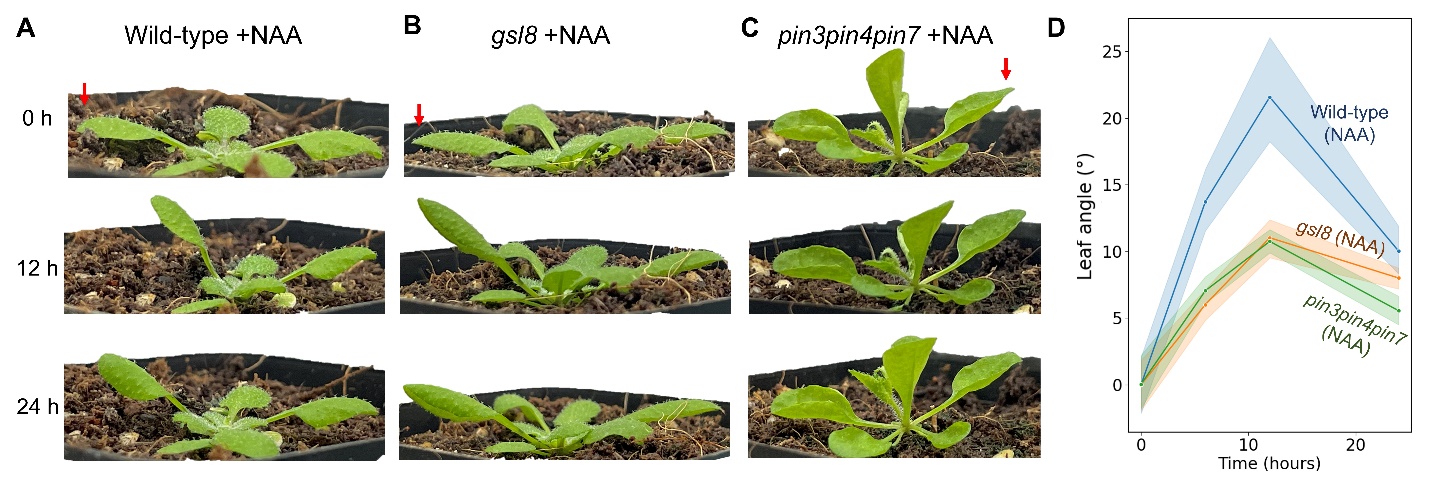
**

**Fig. S2: Hyponasty response in wild-type, *gsl8* and *pin3pin4pin7* plants.** The auxin 1-Naphthaleneacetic acid (NAA) was applied to the tips of leaves of 21-day-old plants (arrow). **A-C** Images showing plants at the start of the experiment (0 h) and after 12 and 24 hours. **D** Leaf angle change relative to the angle at 0 h. Shaded areas indicate 95% confidence intervals. N = 12 (*gsl8*), 10 (all other).

**
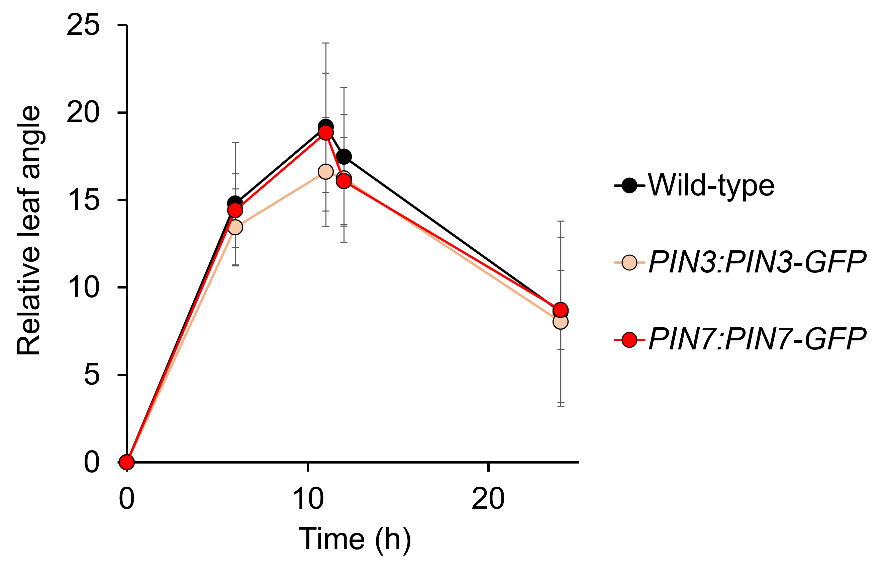
**

**Fig.S3: Hyponasty response of *PIN-GFP* expressing plants.** Leaf angle change after application of auxin to the leaf tip in plants expressing *PIN3:PIN3-GFP* and *PIN7:PIN7-GFP* gene constructs and wild-type plants. Error bars indicate standard deviation. N = 36 (wild-type), 10 (*PIN3:PIN3-GFP*), 6 (*PIN7:PIN7-GFP*).


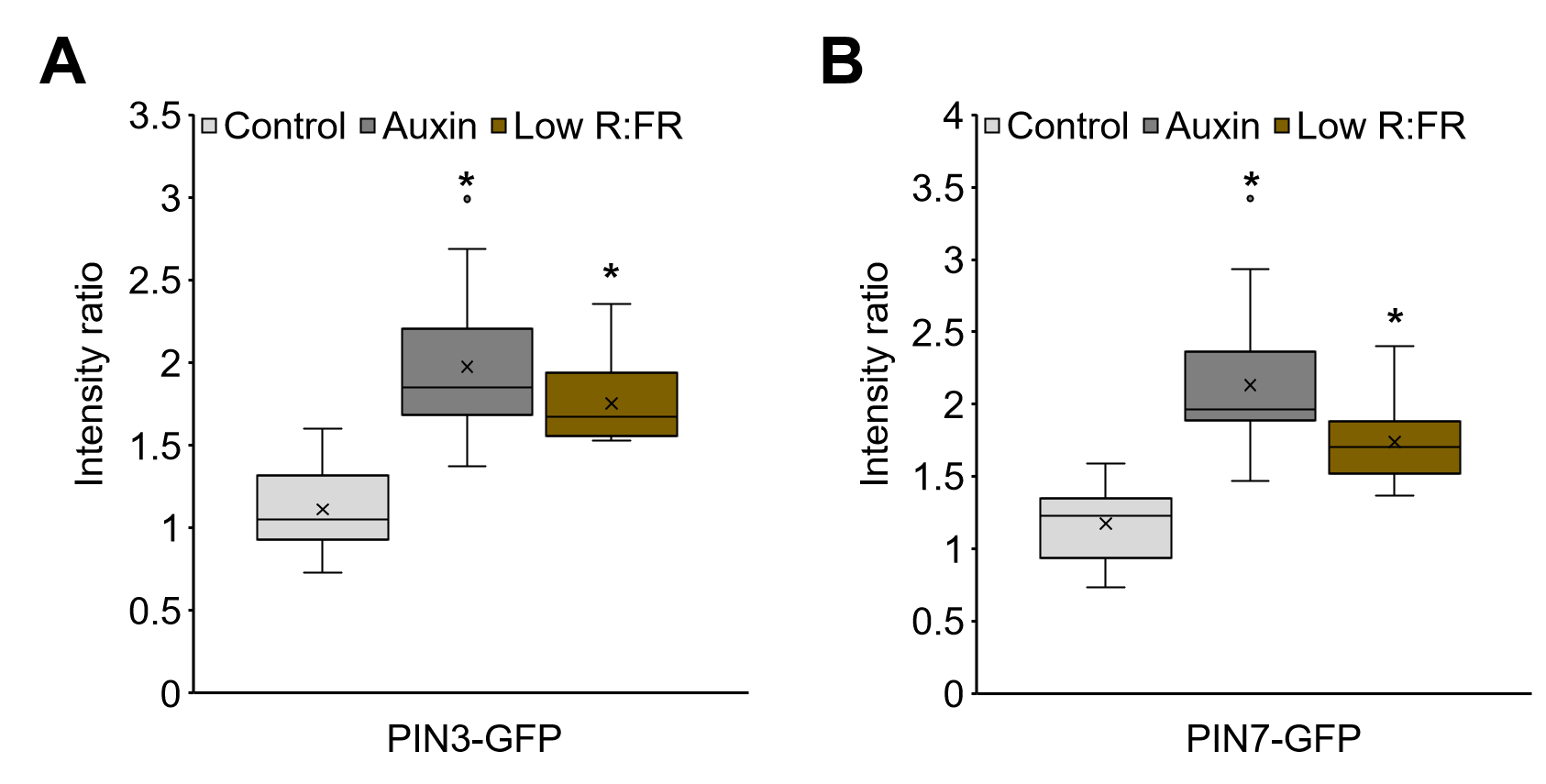


**Fig. S4: Relocation of PIN-GFP fusion proteins during hyponasty response in abaxial midrib epidermis cells.** Hyponasty was induced either by auxin (IAA) application to the leaf tip (auxin) or lowering of the R:FR light ratio at the leaf tip (Low R:FR). Fluorescence intensity of PIN3-GFP (**A**) and PIN7-GFP (**B**) at the plasma membranes longitudinal to the leaf axis divided by the intensity at the plasma membranes transverse to the leaf axis. Asterisks indicate significant difference to wild-type according to Student’s t-test (p < 0.05). Boxes represent quartiles, x represents mean, and bars indicate 95% confidence intervals. N = 20 (control and auxin), 14 (Low R:FR).
